# Coral calcification responses to the North Atlantic Oscillation and coral bleaching in Bermuda

**DOI:** 10.1371/journal.pone.0241854

**Published:** 2020-11-11

**Authors:** Travis A. Courtney, Theodor Kindeberg, Andreas J. Andersson

**Affiliations:** 1 Scripps Institution of Oceanography, University of California, San Diego, California, United States of America; 2 Department of Biology, Lund University, Lund, Sweden; University of Bologna, ITALY

## Abstract

The North Atlantic Oscillation (NAO) has been hypothesized to drive interannual variability in Bermudan coral extension rates and reef-scale calcification through the provisioning of nutritional pulses associated with negative NAO winters. However, the direct influence of the NAO on Bermudan coral calcification rates remains to be determined and may vary between species and reef sites owing to implicit differences in coral life history strategies and environmental gradients across the Bermuda reef platform. In this study, we investigated the connection between negative NAO winters and Bermudan *Diploria labyrinthiformis*, *Pseudodiploria strigosa*, and *Orbicella franksi* coral calcification rates across rim reef, lagoon, and nearshore reef sites. Linear mixed effects modeling detected an inverse correlation between *D*. *labyrinthiformis* calcification rates and the winter NAO index, with higher rates associated with increasingly negative NAO winters. Conversely, there were no detectable correlations between *P*. *strigosa* or *O*. *franksi* calcification rates and the winter NAO index suggesting that coral calcification responses associated with negative NAO winters could be species-specific. The correlation between coral calcification rates and winter NAO index was significantly more negative at the outer rim of the reef (Hog Reef) compared to a nearshore reef site (Whalebone Bay), possibly indicating differential influence of the NAO as a function of the distance from the reef edge. Furthermore, a negative calcification anomaly was observed in 100% of *D*. *labyrinthiformis* cores in association with the 1988 coral bleaching event with a subsequent positive calcification anomaly in 1989 indicating a post-bleaching recovery in calcification rates. These results highlight the importance of assessing variable interannual coral calcification responses between species and across inshore-offshore gradients to interannual atmospheric modes such as the NAO, thermal stress events, and potential interactions between ocean warming and availability of coral nutrition to improve projections for future coral calcification rates under climate change.

## Introduction

For millions of years, calcification by scleractinian corals has accounted for the majority of the growth and maintenance of tropical to subtropical shallow-water coral reef structures [1–3], which currently sustain approximately 10% of humanity with critical ecosystem services including shoreline protection, fisheries provisioning, and cultural significance [4–6]. The current range of optimal environmental conditions have limited coral dominated reef systems to approximately just 0.2% of the global oceans [7, 8]. Within this limited geographic range, considerable variation in coral calcification rates have been observed between species owing to varying ecological traits and within species owing to gradients in the environmental drivers of calcification through space and time [9]. However, ocean warming, acidification, eutrophication, and deoxygenation currently threaten the capacity for corals and coral reefs to maintain their calcium carbonate structures [9, 10]. Understanding the drivers of variability in calcification rates over broad spatial and temporal scales is therefore critical to inform effective management and conservation strategies [10]. Calcification rate time series data can be quantified from the product of linear extension and skeletal density of annual skeletal growth bands to evaluate the potential environmental drivers of coral calcification for many massive coral taxa [9, 11–14]. The high latitude coral reefs of Bermuda were chosen as the focus for this study because they contain coral taxa with observable growth bands and a naturally variable environment that may have conferred an increased resiliency of Bermudan coral communities to environmental change relative to their more southerly conspecifics [15–17].

Relatively few species of scleractinian corals occupy the high latitude coral reefs of Bermuda, but there are nonetheless considerable differences in calcification rates by up to an order of magnitude between species [18]. Within species, extension rates for *Diploria labyrinthiformis*, *Porites astreoides*, and *Pseudodiploria strigosa* have been observed to vary across the Bermuda platform with a tendency towards more rapid extension rates in nearshore environments relative to rim reef environments, which may be due to increased temperature extrema, elevated nutrition, or reduced wave activity closer to shore [19–21]. Further variability in *D*. *labyrinthiformis* and *P*. *astreoides* extension rates have been observed across depth gradients in Bermuda with lower extension rates observed for corals residing in deeper waters relative to shallower locations likely owing to reduced light intensity at deeper depths [20]. At the regional spatial scale, Bermudan coral growth rates are generally lower than their Caribbean conspecifics (e.g., *Diploria labyrinthiformis*, *Pseudodiploria strigosa*, *Orbicella franksi*, *Porites astreoides*, and *Scolymia cubensis*), which has been attributed to the on average cooler waters and reduced light intensity at higher latitudes [18–23].

Coral calcification rates in Bermuda have also been found to vary considerably over time. For example, *P*. *astreoides* and *P*. *strigosa* calcification rates have been shown to vary on diel time scales in association with the beneficial effects of light intensity on calcification rates [24]. *Diploria labyrinthiformis* and *P*. *astreoides* calcification rates were observed to vary on seasonal time scales primarily due to the positive effects of warmer sea surface temperature (SST) on calcification rates [16]. *Diploria labyrinthiformis*, *P*. *strigosa*, and *Montastraea cavernosa* extension rates and skeletal densities have been further observed to vary on interannual time scales in apparent synchrony with the North Atlantic Oscillation (NAO) [19, 25–28]. The NAO refers to the variability in the atmospheric pressure gradient observed at sea level between the subtropical high pressure system near the Azores and the arctic low pressure system near Iceland that drives climatic and oceanographic variability across the North Atlantic Ocean [29–32]. Notably, winters with a negative NAO index (i.e., hereafter referred to as negative NAO winters), are generally characterized by intensified water column mixing that entrain cold, nutrient-rich waters that drive enhanced phytoplankton blooms and community composition shifts in the North Atlantic subtropical gyre near Bermuda [29–34]. Elevated extension rates and reduced skeletal densities have been ascribed to increased coral nutrition (e.g., particulate organic matter [35]) coinciding with cooler SST during negative NAO winters [19, 25–28] with coral calcification rates assumed to remain approximately constant owing to the offsetting increases in extension rates and decreases in skeletal density [27]. However, summertime coral reef net ecosystem calcification on the Bermuda platform was found to increase following negative NAO winters [36] and food availability was positively correlated with increasing Bermudan coral calcification rates on seasonal time scales [16] suggesting negative NAO winters may also benefit Bermudan coral calcification rates.

The primary objective of this study was to test the hypothesis that negative NAO winters increase annual coral calcification rates across the Bermuda platform, which may be driven by a postulated increase in coral nutrition stimulating coral calcification rates on the reef [37]. We further hypothesize that negative NAO winters differ between species owing to differences in life history strategies, which can modulate calcification responses to environmental conditions [38]. Finally, we hypothesize that coral calcification rates and their responses to negative NAO winters vary across the platform, which may be due to the differential influence of the NAO interacting with environmental gradients across the Bermuda platform. To test these hypotheses, annual calcification rates from *Diploria labyrinthiformis*, *Orbicella franksi*, and *Pseudodiploria strigosa* coral cores collected across the Bermuda platform were analyzed with respect to the winter NAO phase. However, it is important to emphasize that the data presented here only assess the links between Bermudan coral calcification rates and the winter NAO index, and not the actual interannual nutritional availability on the reef, which remains a working hypothesis of the potential connection between the winter NAO index and coral calcification rates.

## Methods

### Species and site descriptions

**Fig 1:**
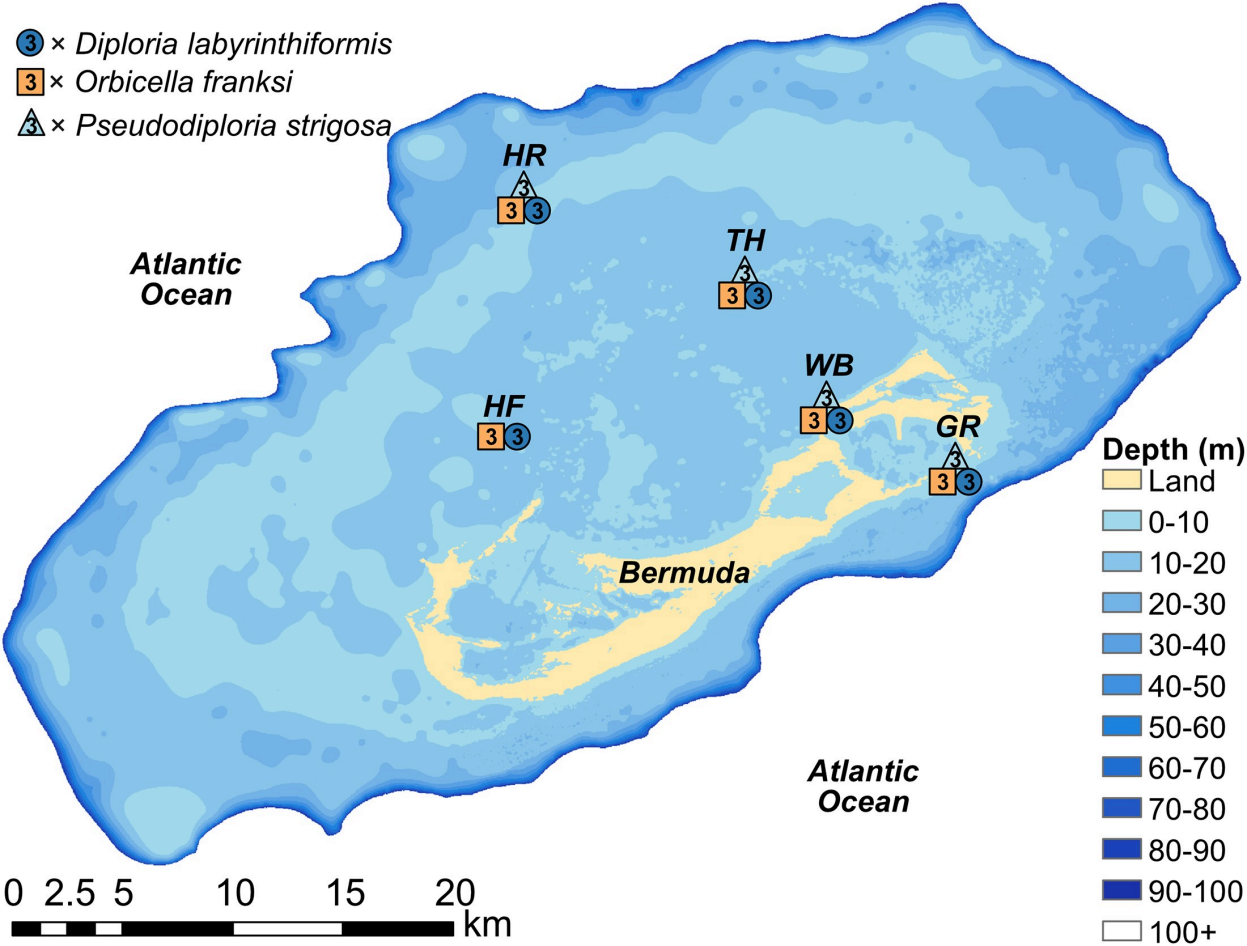
Map of coral coring locations across the Bermuda platform. Colored shapes correspond to locations where three cores were collected from the respective species at each of the following reef sites: HR = Hog Reef; TH = Three Hill Shoals; WB = Whalebone Bay; GR = Gurnet Rock; and HF = Halfway Flat. Bathymetry data are from [45].

*Diploria labyrinthiformis*, *O*. *franksi*, and *P*. *strigosa* were selected for coring in this study because they form massive skeletal morphologies with documented density bands [21, 39–41], use both tentacle and mucous-based feeding strategies [42], and represent some of the dominant reef-building corals in Bermuda [18]. Additionally, *D*. *labyrinthiformis* and *P*. *strigosa* are both considered as having stress-tolerant life histories whereas *O*. *franksi* is considered as having a generalist life history strategy, which suggests that their responses to environmental conditions may differ [38]. All coring sites were within the boundaries of the rim reefs surrounding Bermuda along a gradient of more oceanic to terrestrial proximity (Fig 1). The North Lagoon coring locations include an outer rim reef site (Hog Reef), two centrally located lagoon patch reef locations (Halfway Flat and Three Hill Shoals), and a nearshore reef site (Whalebone Bay, Fig 1). The south shore Gurnet Rock coring site is proximal to the open ocean and is also located at the tidal outflows of higher turbidity Castle Harbour inshore waters [43, 44] suggesting it may experience a mixture of nearshore and oceanic characteristics (Fig 1).

Limited observations support a gradient of decreasing seasonal temperature extrema, decreasing coral nutrition, and increasing wave energy from the nearshore to lagoon to rim reef locations [16, 19–21, 37, 43, 46–49]. For example, in situ seawater temperature ranges in 2006 decreased slightly from nearshore to rim reef locations with observations of 15.6–30.1°C for Whalebone Bay, 17.3–29.6°C for Crescent Reef (located near Halfway Flat), 17.1–29.4°C for Three Hill Shoals, and 17.9–29.0°C for Hog Reef [46]. Recorded salinity values have been reported to be relatively constant at 36.5 ppt across the majority of the Bermuda platform [50]. The Bermuda Inshore Waters Investigation provides some evidence for decreasing coral nutrition from nearshore to lagoon waters with higher nitrogen content of particulate matter (Great Sound: 4.0 μmol l^–1^ vs. North Lagoon: 2.8 μmol l^–1^), higher phosphorous content of particulate matter (Great Sound: 0.11 μmol l^–1^ vs. North Lagoon: 0.06 μmol l^–1^), and higher chlorophyll α concentrations (Great Sound: 0.78±0.37 μg l^–1^ vs. North Lagoon: 0.43±0.19 μg l^–1^) observed at the more nearshore Great Sound sampling location relative to the North Lagoon sampling location [49]. Higher turbidity was also observed for the more nearshore reef sites compared to the rim reef sites, suggesting a greater abundance of suspended particulate matter available for coral heterotrophy at nearshore relative to rim reef sites [37]. Mean flow speeds (*ū*) and turbulence intensities (*q*) on a calm day were lowest nearshore at Tynes Bay (*ū* = 1.64–2.03 cm s^–1^, *q* = 0.85–1.09 cm s^–1^), higher in the central lagoon at Crescent Reef (*ū* = 2.22–2.52 cm s^–1^, *q* = 1.03–1.11 cm s^–1^), and highest in the rim reef at Hog Reef (*ū* = 5.58–8.42 cm s^–1^, *q* = 2.49–4.49 cm s^–1^), which in part supports a gradient of increasing wave energy from nearshore to lagoon to rim reef locations [37]. We caution that further measurements across space and time are necessary to more strongly elucidate the persistence and variability in environmental parameters across the Bermuda platform while the data presented here nonetheless provide some evidence to support the presence of environmental gradients from the nearshore to rim reef environments.

### Sample collection

All coral cores were collected in August 2016 using a pneumatic drill with 5-cm diameter wet concrete diamond core drill bits powered by a boat-based air compressor or underwater diving cylinders. Following extraction, cement plugs were placed in each colony and sealed with underwater epoxy to promote coral recovery. Each coral colony was selected for coring based on similar colony heights of approximately 30 cm to limit potential ontogenetic effects on growth rates [39–42], within a mean (±SD) depth of 4.5±1.8 m to limit depth effects [20], and with seemingly healthy appearances to reduce the effects of other potential confounding variables on growth rates [43]. Each coral core was collected along the vertical growth axis up to 30 cm in length from three replicate colonies of *D*. *labyrinthiformis* (n = 15), *P*. *strigosa* (n = 12), and *O*. *franksi* (n = 15) from four to five distinct reef locations for a total of 42 cores from 42 distinct coral colonies (Fig 1). Regrettably, fewer cores were collected from *P*. *strigosa* because no colonies meeting our search criteria were observed at the central platform reef site (Fig 1). The Government of Bermuda Department of Environment and Natural Resources is acknowledged for permitting collection and approving exports of the coral cores under license number 2015–05–26–46.

### Quantification of linear extension, skeletal density, and calcification

Coral cores and density reference materials from the laboratory of Dr. Karl Castillo at the University of North Carolina at Chapel Hill were imaged via computed tomography (CT) with a GE Lightspeed VCT at San Diego Imagery. Individual cores and density standards were visualized as the mean voxel intensity of a ~3.00 mm thick digital slice using standardized window widths and window levels in the software *Horos* (version 2.4.1) and exported as an image for measuring growth rates in *Coral XDS* [51]. To quantify density from CT image intensity following established methods [52–55], linear models between known density and CT intensity of the density reference materials for three independent scans were derived using the function *lm* in the statistical package *R* version 4.0.2 [56]. Transects were analyzed along the major growth axis of individual polyps to quantify mean extension and intensity of subsequent high and low intensity bands using the half range mode and converted to skeletal density using the density calibration curves for each scan. Annual extension was determined as the sum of extension for adjacent high and low intensity bands while annual skeletal density was determined as the weighted average of skeletal densities for adjacent high and low skeletal density bands to account for any potential differences in extension rates between bands. Annual calcification rates (g cm^–2^ yr^–1^) were determined from the product of annual extension (cm yr^–1^) and skeletal density (g cm^–3^) [11, 13, 14, 57]. The 2016 growth bands were incompletely formed due to the timing of collection and were removed from the data prior to subsequent analyses of annual calcification rates.

### Time series construction and pointer year analyses

To visualize temporal trends, mean chronologies (±95% confidence intervals) were developed for the annual linear extension, skeletal density, and calcification data from each coral core (S2 Fig) and the monthly winter NAO (DJFM = December, January, February, and March) index from the NOAA National Weather Service (i.e., 1950–2015, URL: https://www.cpc.ncep.noaa.gov/products/precip/CWlink/pna/nao.shtml) using the function *MeanCI* in the *R* package *DescTools* [58]. A pointer year analysis was used to test whether individual negative NAO winters corresponded to detectable deviations in annual coral linear extension, skeletal density, and calcification. In this study, pointer years were defined as individual years that exhibited ±10% positive or negative growth rate anomalies relative to the previous year in at least 75% of the cores for each year [59, 60] using the *R* package *dplR* [61]. Pointer years with less than one third of the full series of cores from each species were excluded to avoid potential false detection of growth rate anomalies based on a limited number of cores. The pointer year analyses were conducted separately for all cores collected from each species (e.g., *D*. *labyrinthiformis* [n = 15], *P*. *strigosa* [n = 12], and *O*. *franksi* [n = 15]) to test for species-level effects of negative NAO winters on annual linear extension, skeletal density, and calcification.

### Linear mixed effects modeling

Linear mixed effects modeling was used to detect the fixed effects of winter NAO index, species, site, and the interactions between the winter NAO index and species or site on annual calcification rates for the duration of the NAO index (i.e., 1950–2015) using the *R* package *nlme* [62]. This analytical approach was used to account for nesting of calcification rates within each core and data imbalances generated by variable lengths of core time series data with the capacity to detect small trends in coral calcification rates following established methods [53, 55, 63]. Briefly, each coral core was treated as an individual sampling unit with annual calcification rates nested within each core. Random effects were incorporated to allow the estimated slopes and intercepts to vary by core. A stepwise modeling approach building from simple null models to more complex models utilizing random and fixed effects was followed with minimized AIC values used to select the optimal model. Significance tests of the main effects and interactions for the optimal model were evaluated with ANOVA (Type III) in the *anova*.*lme* function in *R* package *nlme* [62]. Simple slopes for site and species that incorporate the main effects and interactions and Tukey Honest Significant Difference (HSD) pairwise comparisons of species and site were determined using the function *emtrends* in *R* package *emmeans* [64].

## Results

### Density calibrations

The measured densities of coral core standards were strongly correlated with CT scan intensity for three independent scans (Scan 1: Slope = 0.019±0.002, adjusted R^2^ = 0.92, p<0.001; Scan 2: Slope = 0.018±0.001, adjusted R^2^ = 0.97, p<0.001; Scan 3: Slope = 0.0205±0.0009, adjusted R^2^ = 0.98, p<0.001). The 95% confidence intervals of the predicted mean skeletal densities for all cores based on these regressions generates a mean calculated skeletal density uncertainty of ±0.06 g cm^–3^ (S1 Fig).

### Mean chronologies and pointer years

**Fig 2:**
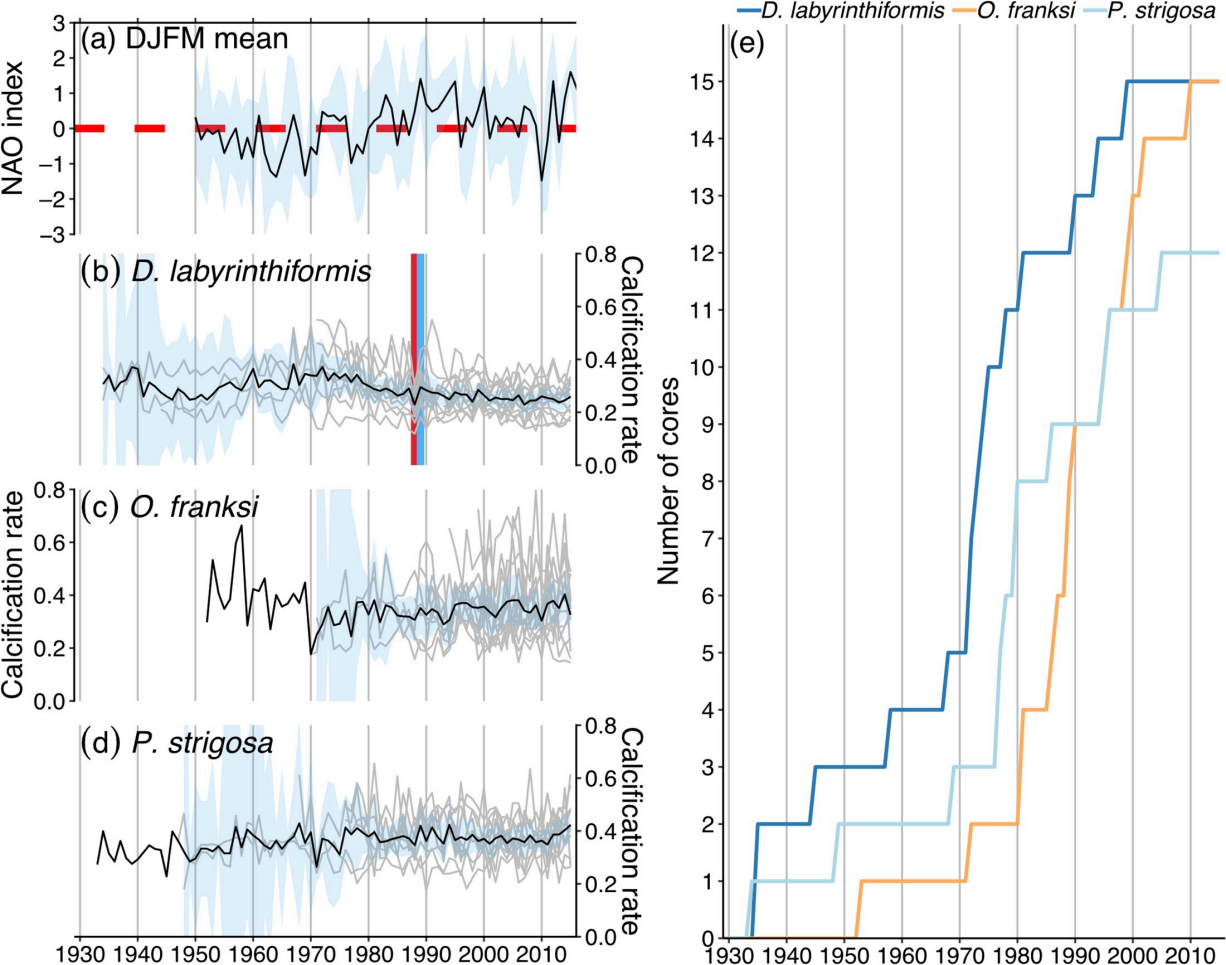
Mean winter NAO index and coral core calcification chronologies. (a) Black line indicates mean of NAO index for DJFM with shaded ±95% confidence intervals in light blue and dashed-red line indicating zero. (b,c,d) Gray lines indicate calcification rates (g cm^–2^ yr^–1^) for individual coral cores and the black lines with light blue shading represent mean calcification rates ±95% confidence intervals for each species. The vertical red line indicates the negative pointer year and the vertical light blue line indicates the positive pointer year. (e) Lines indicate the number of cores with calcification rate data for the respective year from each species.

The winter NAO index exhibited interannual variability with a tendency towards more negative phases from approximately 1950–1980 and more positive phases thereafter with the exception of a notably negative NAO winter in 2010 (Fig 2a). There was a high degree of variability in calcification rates between cores and years that tended to increase towards the base of the time series (Fig 2b–2d) as the number of cores with growth rate data for the respective year declined (Fig 2e). No apparent long-term calcification trends were observed except for *D*. *labyrinthiformis*, which appeared to be slightly elevated from approximately 1950–1985 (Fig 2b–2d). Coral core chronologies with respect to the mean annual extension, skeletal density, and calcification data for each species reflected similar trends with calcification appearing to more closely follow patterns in linear extension than skeletal density (S2 Fig). There was a recorded negative pointer year in 1988 (100% of cores exhibited negative growth relative to the previous year) and a positive pointer year in 1989 (83% of cores exhibited positive growth relative to the previous year) in the annual calcification time series data for *D*. *labyrinthiformis* (Fig 2). No other pointer years were detected in the linear extension, skeletal density, or calcification rate data (S2 Fig).

### Linear mixed effects modeling

**Fig 3:**
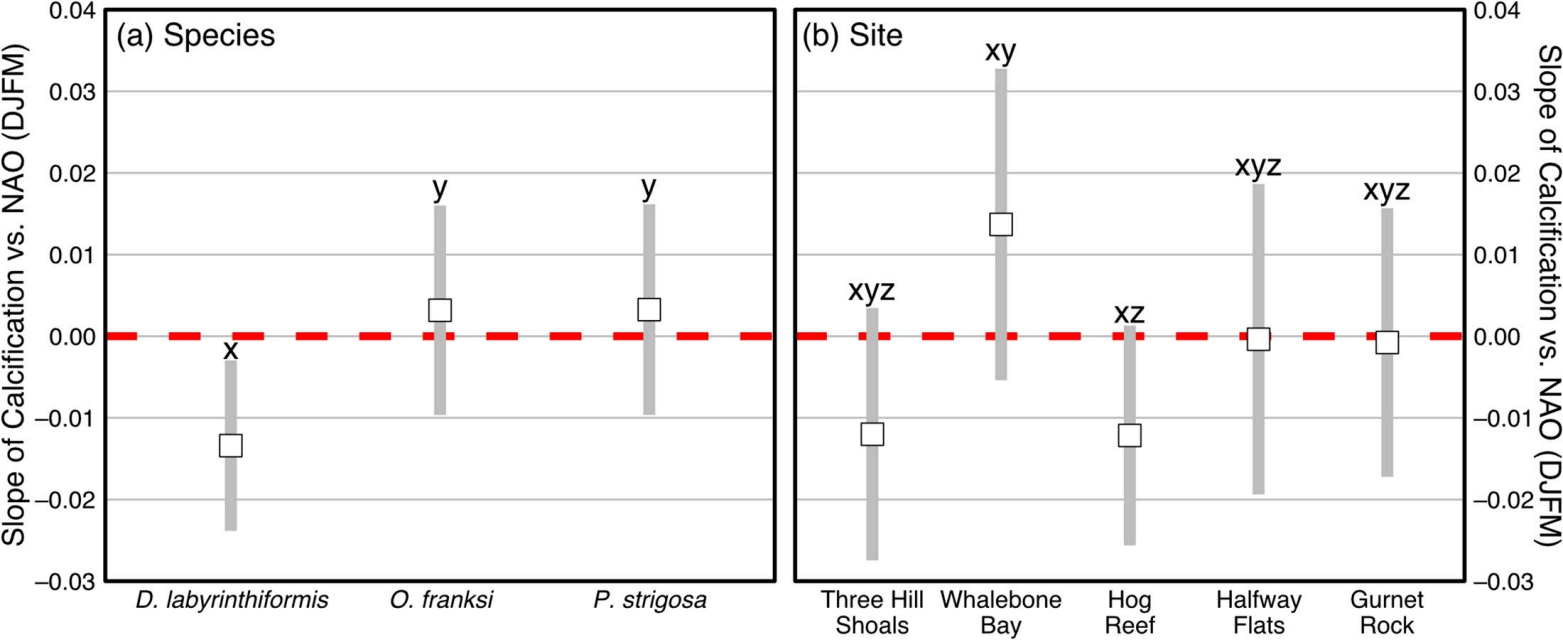
Simple slopes calculated from linear mixed effects modeling of calcification vs. NAO (DJFM) index main effects and interactions for each coral species (a) and site (b). Squares represent mean slope value with shaded bars representing ± 95% confidence intervals and the red dashed line indicates zero slope. The letters overlying each confidence interval represents statistical significance from post-hoc Tukey Honest Significant Difference (HSD) tests between species (a) and sites (b). In this notation, slopes identified by the same letters are not significantly different from each other and slopes with different letters are significantly different from each other.

The random slopes and intercepts model with winter NAO index, species, site, interaction of winter NAO index with species, and interaction of winter NAO index with site as fixed effects was selected as the best model to fit the annual calcification rates (Table 1). Type III ANOVA revealed that species (p = 0.0002) and the interactions of winter NAO index with species (p = 0.0130) and site (p = 0.0319) were significant predictors of calcification rates while the main effects of winter NAO index (p = 0.0990) and site (p = 0.0960) were not (Table 2). The simple slope (±SE) for calcification per unit winter NAO index was negative for *D*. *labyrinthiformis* (–0.013±0.004 g cm^–2^ per NAO) and significantly lower than the slopes for *O*. *franksi* (0.003±0.005 g cm^–2^ yr^–1^ per NAO) and *P*. *strigosa* (0.003±0.005 g cm^–2^ yr^–1^ per NAO), which exhibited no detectable trends or differences between each other (Fig 3a, Table 3). Similarly, there were no detectable trends in simple slopes in calcification with respect to the NAO across all sites (i.e., 95% confidence intervals overlap zero), but simple slopes at Hog Reef were significantly more negative than for Whalebone Bay (Fig 3b, Table 3).

**Table 1:** Summary of AIC values for stepwise calcification rate model construction with varying random and fixed effects.

Model	AIC
calc ~ 1	-2657.378
calc ~ 1, random: ~ 1|coral	-3491.836
calc ~ NAO	-2655.428
calc ~ NAO, random: ~ 1|coral	-3494.233
calc ~ NAO, random: ~ NAO|coral	-3498.068
calc ~ NAO + species, random: ~ NAO|coral	-3506.648
calc ~ NAO + species + NAO:species, random: ~ NAO|coral	-3510.373
calc ~ NAO + site, random: ~ NAO|coral	-3495.665
calc ~ NAO + site + NAO:site, random: ~ NAO|coral	-3495.265
calc ~ NAO + species + site, random: ~ NAO|coral	-3505.946
calc ~ NAO + species + NAO:species + site, random: ~ NAO|coral	-3509.756
calc ~ NAO + site + NAO:site + species, random: ~ NAO|coral	-3507.030
**calc ~ NAO + species + site + NAO:species + NAO:site, random: ~ NAO|coral**	**-3511.583**

Coral calcification rates are represented by “calc”, mean phase of the winter NAO is represented by “NAO”, cored species is represented by “species”, coring site is represented by “site”, and individual coral cores are represented by “coral.” The bold model had the lowest AIC value and was selected as the best model to fit the annual calcification rate data. Models without random effects were parameterized using the function *lm* in base *R* [56] and models with random effects were parameterized using the function *lme* in package *nlme* [62].

**Table 2:** Summary of ANOVA (Type = III) results of the best model to fit the annual calcification rate data.

predictor	df_numerator_	df_denominator_	F-value	p-value
**(Intercept)**	**1**	**1503**	**192.148**	**<0.0001**
NAO	1	1503	2.725	0.0990
**species**	**2**	**35**	**11.395**	**0.0002**
site	4	35	2.144	0.0960
**NAO:species**	**2**	**1503**	**4.356**	**0.0130**
**NAO:site**	**4**	**1503**	**2.649**	**0.0319**

“Predictor” represents the model parameter, “df_numerator_” and “df_denominator_” represents numerator and denominator degrees of freedom, respectively, “F-value” represents the F statistic for each predictor, and each “p-value” less than α = 0.05 is in bold text.

**Table 3:** Summary of Tukey Honest Significant Difference (HSD) pairwise comparisons of species and site for the best model to fit the annual calcification rate data.

Contrast	Estimate	SE	t-ratio	p-value
***D*. *labyrinthiformis—O*. *franksi***	**-0.0165798**	**0.00689**	**-2.408**	**0.0427**
***D*. *labyrinthiformis—P*. *strigosa***	**-0.0166635**	**0.00683**	**-2.439**	**0.0394**
*O*. *franksi—P*. *strigosa*	-0.0000837	0.00776	-0.011	0.9999
Gurnet Rock—Halfway Flat	-0.000403	0.00979	-0.041	0.9999
Gurnet Rock—Hog Reef	0.011389	0.00821	1.387	0.6360
Gurnet Rock—Three Hill Shoals	0.011235	0.00875	1.284	0.7010
Gurnet Rock—Whalebone Bay	-0.014462	0.00978	-1.479	0.5762
Halfway Flat—Hog Reef	0.011792	0.00916	1.287	0.6995
Halfway Flat—Three Hill Shoals	0.011638	0.00950	1.225	0.7369
Halfway Flat—Whalebone Bay	-0.014060	0.01061	-1.325	0.6755
Hog Reef—Three Hill Shoals	-0.000155	0.00791	-0.020	0.9999
**Hog Reef—Whalebone Bay**	**-0.025852**	**0.00902**	**-2.867**	**0.0341**
Three Hill Shoals—Whalebone Bay	-0.025697	0.00954	-2.695	0.0552

“Contrast” represents the pairwise comparisons, “Estimate” represents the mean difference between pairwise comparisons, “SE” represents the standard error of the difference between pairwise comparisons, “t-ratio” represents the t-ratio for each pairwise comparison, and each “p-value” less than α = 0.05 for each pairwise comparison is in bold text.

## Discussion

The negative correlation between *D*. *labyrinthiformis* calcification rates and winter NAO index partially supports the hypothesis that stronger negative winter NAO phases influence annual Bermudan coral (Fig 3a) calcification rates [36]. This extends the established links between coral extension rates and skeletal densities [19, 25, 26, 28] to include enhanced coral calcification rates associated with negative NAO winters, which are characterized by cooler SST, elevated nutrients, and enhanced phytoplankton blooms in offshore waters [29–34]. However, there were no detectable correlations between *O*. *franksi* or *P*. *strigosa* calcification rates and the winter NAO index (Fig 3a), which may suggest that calcification responses to NAO linked changes vary by species. While we cannot unequivocally rule out the possibility that the increased coverage of calcification time series data in earlier years preferentially enabled detection of the winter NAO index impacts on *D*. *labyrinthiformis* calcification rates, all three species have calcification rates overlapping with nearly the entire 1950–2015 winter NAO index suggesting we could still expect to detect any real correlations with the NAO (Fig 2). Negative NAO winters may indeed be more beneficial to *D*. *labyrinthiformis* calcification rates than *O*. *franksi* and *P*. *strigosa* (Fig 3a), but the mechanisms for this potential difference such as potential provisioning of nutritional pulses to the Bermudan corals during negative NAO winters remains to be elucidated. For example, heterotrophic status may be more similar for *D*. *labyrinthiformis* and *P*. *strigosa* on the basis of similar suspension feeding rates [65] and tissue thickness (S1 Table) that were greater than for *O*. *franksi*. However, *O*. *franksi* and *P*. *strigosa* exhibited statistically similar nitrogen uptake of particulate matter in a controlled feeding experiment [37] and had similar calcification responses to the winter NAO index in this study (Fig 3a). Future studies are needed to more firmly establish the mechanisms for the species-specific calcification responses to the winter NAO observed herein (Fig 3a). Critically, such studies would further elucidate the species-specific effects of heterotrophy on Bermudan coral calcification rates and the inshore-offshore coupling in phyto- and zooplankton biomass with respect to the NAO.

Spatial gradients in nutrition with respect to coral growth rates have long been hypothesized for the Bermuda platform [19, 21, 37]; however, environmental data to support the higher concentration of available coral nutrition on nearshore reef sites is sparse (e.g., see Species and site descriptions section) and warrants further investigation [16, 19–21, 37, 43, 46–49]. While no individual sites had detectable correlations between calcification and the winter NAO index, the correlation for the most outer rim reef coring site, Hog Reef, was significantly more negative than for the most nearshore coring site, Whalebone Bay (Fig 3, Table 3). There is therefore some tentative evidence that the negative NAO index may be more beneficial to coral calcification rates at the outer rim reefs than nearshore reefs. This may be due to the stronger influence of postulated nutritional pulses in negative NAO winters along the rim reef compared to nearshore because of lower background concentrations of nutrition at the former [16, 43, 46–48]. However, further research is needed to establish the mechanisms for any potential spatial differences in the response of coral clacification rates to the winter NAO index across the Bermuda platform. For example, site was not a significant predictor of calcification rates in this study (p = 0.0960, Fig 3b, Table 2), but previous studies with greater sample sizes (e.g., n = 7–18; [21]; n = 1–34 [19]) were able to detect elevated extension rates at sites more proximal to shore relative to sites closer to the outer rim reef [19, 21]. It is important to note that while calcification rates may respond more strongly to environmental conditions than linear extension [66], uncertainties in the measurement of calcification rates may be greater than for linear extension [67]. Therefore, an increased sampling size beyond the three corals per species per site in this study may be needed to further elucidate site-level differences in Bermudan coral calcification rates and their responses to the winter NAO index.

In contrast to the overall correlation between *D*. *labyrinthiformis* calcification rates and winter NAO, the identified negative pointer year in 1988 (winter NAO index = 0.48) and positive pointer year in 1989 (winter NAO index = 1.41) (Fig 2b), do not support enhanced coral growth rates associated with negative NAO winters. Instead, the reduced calcification rates for 100% of *D*. *labyrinthiformis* cores in 1988 coincided with the year of the first reported coral bleaching event in Bermuda [68] and suggest that the 1988 bleaching event impacted *D*. *labyrinthiformis* calcification rates more negatively than *O*. *franksi* and *P*. *strigosa*. The positive pointer year in 1989 and absence of any noticeable post-bleaching depression of annual calcification rates [69, 70] in the time series data (Fig 2b) suggests a rapid recovery in *D*. *labyrinthiformis* calcification rates. Furthermore, high density skeletal growth anomalies have been associated with coral bleaching events [70–79], but no positive pointer years were detectable in the skeletal density data of this study (S2 Fig). While subsequent minor coral bleaching events have occurred throughout the Bermuda platform in the years following the 1988 bleaching event [15], there were no detectable impacts of these additional events on calcification rates in the pointer year analyses of this study (Fig 2b–2d). The single negative pointer year observed in 1988 suggests that the coincident coral bleaching event may have been the most intense bleaching event on record. Further research is needed to determine whether this is due to potential differences in the magnitude of subsequent marine heat waves in Bermuda or potential long-term acclimatization of Bermudan corals following the 1988 bleaching event.

**Fig 4:**
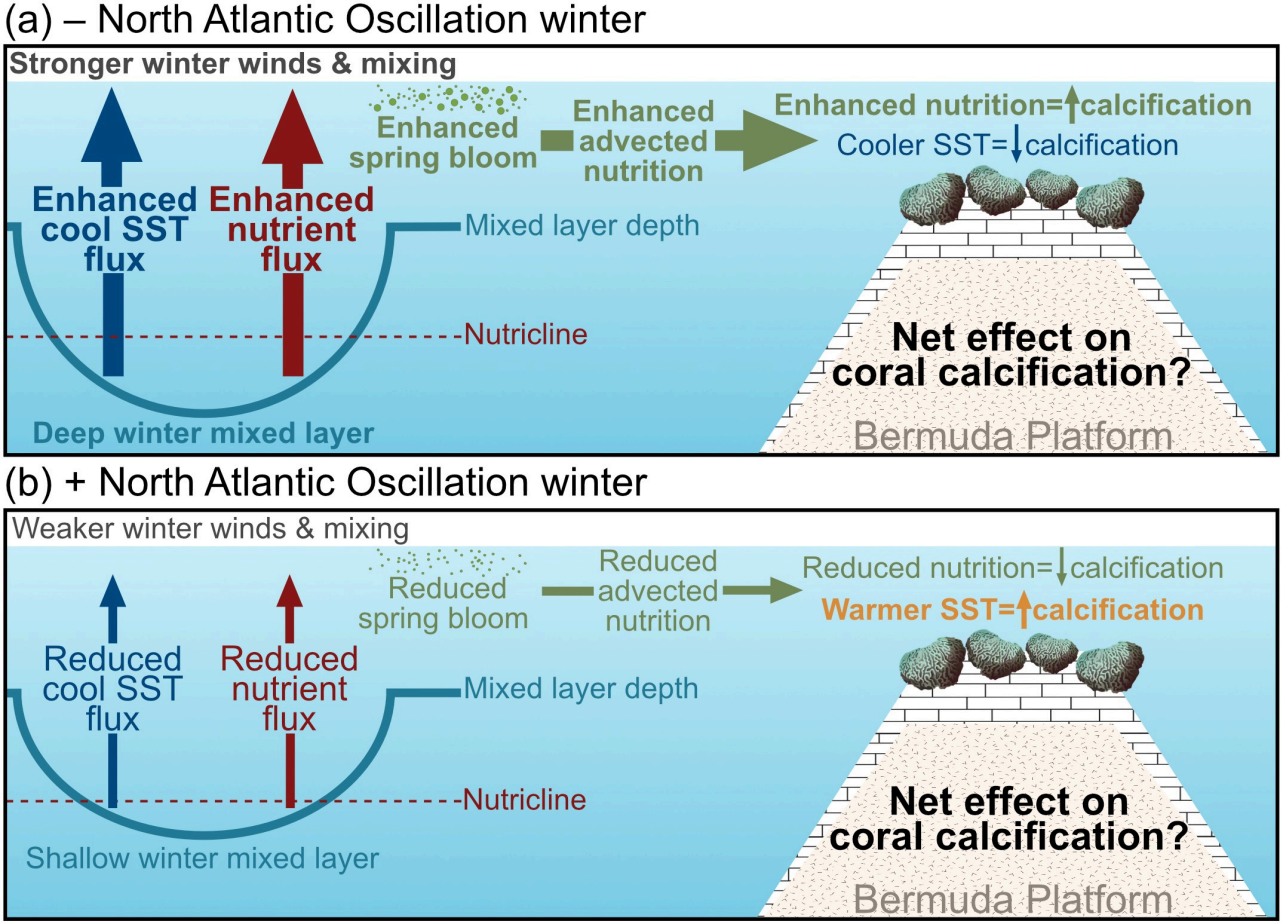
How do changing winter sea surface temperatures (SST) influence high latitude coral calcification rates through the potential interannual tradeoffs between the beneficial effects of warmer SST and enhanced coral nutrition on calcification rates? The figure is modeled after [36] and the brain coral illustration (not drawn to scale) is courtesy of the integration and application network, university of Maryland center for environmental science (ian.umces.edu/symbols/).

The high degree of stability of Bermudan coral communities over recent decades is evidenced by the lack of any long-term declines in coral cover or changes in the coral community composition [15–17] that have been observed for many of the lower-latitude Western Atlantic coral reefs [17, 38, 80–83]. However, it remains unclear whether this stability is owing to Bermudan corals thus far escaping or resisting thermal stress events [16] as unprecedented marine heatwaves have driven extensive coral mortality in other high-latitude coral reef systems with previously stable coral cover [84]. Interestingly, previous work in Bermuda has indicated that the thermal optima for *D*. *labyrinthiformis* [16] and *O*. *franksi* [85] are not currently exceeded by typical summer SST, but that calcification rates under future warming and acidification may become increasingly dependent on the continued availability of adequate nutrition [16, 85]. Warming winters have been hypothesized to benefit high latitude corals through warming calcification rate-limiting winter SST [9, 16]. However, increases in SST (i.e., +0.021°C yr^–1^) have coincided with declines in offshore primary productivity (i.e., –5.6 mg C m^–2^ d^–1^ per year) from 1990–2016 in the offshore waters of Bermuda suggesting these sources of nutrition may decrease with continued warming [86]. The mechanistic links between greenhouse gas emissions and the NAO remain equivocal [29, 31, 87, 88] making it difficult to project how the NAO may interact with climate change to influence offshore primary productivity and Bermudan coral calcification rates. We posit that if warming-induced declines in offshore nutrition persist and they are indeed an important source of Bermudan coral nutrition, then this will negatively affect future Bermudan and other high-latitude coral calcification rates, but the relative balance between the positive effects of winter warming and negative effects of reduced nutrition on calcification rates remains unclear (Fig 4). Meanwhile, the effects of warming on calcification rates in lower latitude corals is hypothesized to be more negative owing to summer SST exceeding coral thermal optima [9] with coinciding declines in nutrition likely to reduce the capacity of corals to resist and recover from the projected increases in coral bleaching events [89–91] (but see also the potential role of nutrients in coral bleaching events [92]). Regardless, reductions in the rates of greenhouse gas emissions under the Paris Agreement remain critical to reduce the probability of frequent and severe coral bleaching events in Bermuda [16] and worldwide [93].

## Conclusions

Collectively the findings of this study highlight the complex interplay between coral species and site on calcification responses associated with the NAO and a thermal stress event. Calcification rates of *D*. *labyrinthiformis* responded most strongly to the winter NAO index and the marine heatwave associated with the 1988 coral bleaching event, while *O*. *franksi* and *P*. *strigosa* exhibited no detectable interannual calcification responses to the NAO or the 1988 bleaching event. Coral calcification responses were more sensitive to negative NAO winters at Hog Reef compared to Whalebone Bay, lending provisional support of potential platform-scale trends in calcification responses to the NAO. In addition to improved Bermudan coral futures under lower CO_2_ emission pathways [16, 85], further research should therefore be conducted to elucidate the mechanisms for the variable calcification responses to the NAO, marine heatwaves, and the potential role of coral nutrition between species and sites in Bermuda and throughout corals’ geographic ranges.

## Supporting information

S1 Fig Coral core standards density curves are plotted for each scan. Each circle represents the mean CT-scan luminance and measured density for each coral standard. Solid black line represents linear regression of measured density vs. CT-scan luminance with shaded gray regions indicating ±95% confidence intervals of regression data.
(TIFF)

S2 Fig Coral core linear extension, skeletal density, and calcification rate time series data are plotted for each species. Gray lines indicate growth measurements for individual coral cores, black lines represent mean coral growth measurements for each species, and shaded blue regions indicate ±95% confidence intervals of the species-level mean time series data. The vertical red line indicates a negative pointer year and vertical light blue line indicates a positive pointer year.
(TIFF)

S1 Table Mean (± standard deviation) core physiological measurements of the entire cored material and the number of cores collected are summarized for all cores from each species and separately for each species from each site. Tissue thickness was measured on the day of collection for each core using Vernier calipers following established methods [94]. Mean coral extension for each core was determined as the mean of the top five years of the coral growth analysis (i.e., 2010–2015) to compare the most recent overlapping growth intervals. Core skeletal densities were determined as the mean skeletal density for the entire cored material to account for variations in skeletal densities between skeletal components. Mean calcification rates for each core were determined as the product of the most recent 5-year mean extension rate and mean core skeletal density [9, 11–14]. Mean annual linear extension, skeletal density, and calcification rates were computed for the entire cored skeletal material to provide context for future physiological studies and carbonate production estimates.
(DOCX)
