# Supplementary figures and images for: Coral calcification responses to the North Atlantic Oscillation and coral bleaching in Bermuda
Source: PLoS One. 2020 Nov 11;15(11):e0241854. doi: 10.1371/journal.pone.0241854 (PMC7657549; doi:10.1371/journal.pone.0241854)

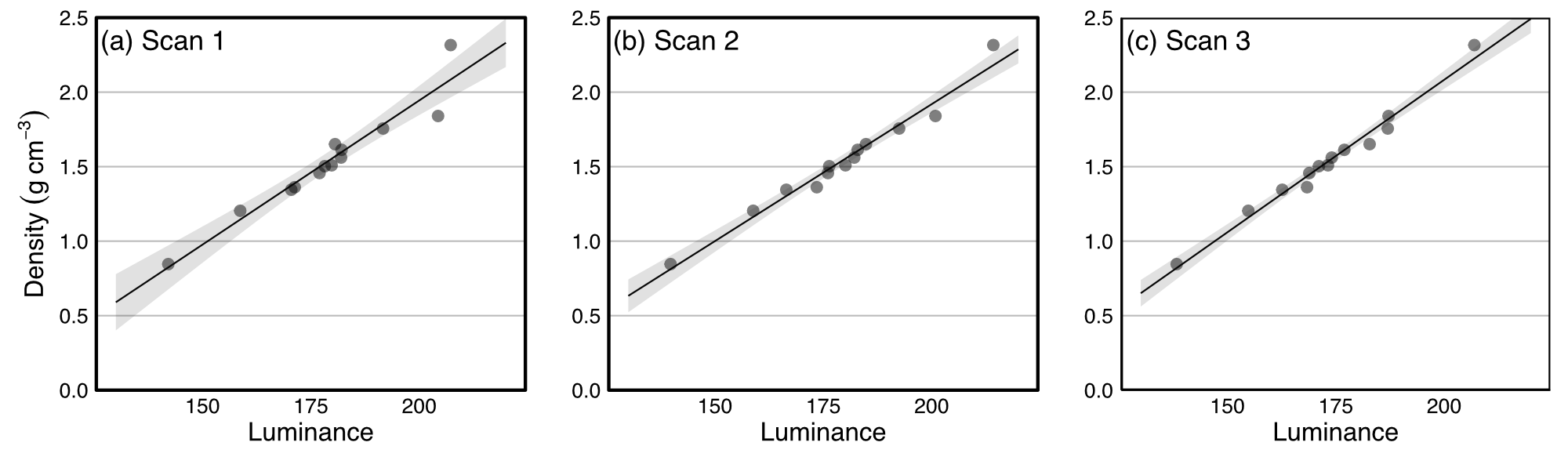

Supplement: S1 Fig — Each circle represents the mean CT-scan luminance and measured density for each coral standard. Solid black line represents linear regression of measured density vs. CT-scan luminance with shaded gray regions indicating ±95% confidence intervals of regression data. (TIFF) [file pone.0241854.s001.tiff]

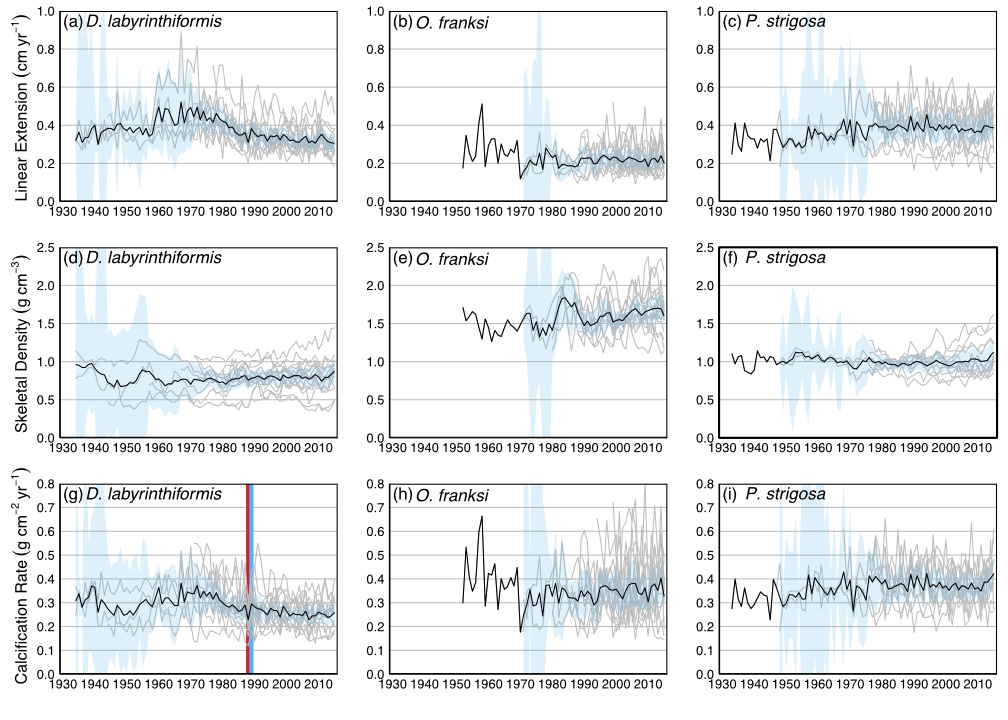

Supplement: S2 Fig — Gray lines indicate growth measurements for individual coral cores, black lines represent mean coral growth measurements for each species, and shaded blue regions indicate ±95% confidence intervals of the species-level mean time series data. The vertical red line indicates a negative pointer year and vertical light blue line indicates a positive pointer year. (TIFF) [file pone.0241854.s002.tiff]
