# Supplementary material for: Coral calcification responses to the North Atlantic Oscillation and coral bleaching in Bermuda
Source: PLoS One. 2020 Nov 11;15(11):e0241854. doi: 10.1371/journal.pone.0241854 (PMC7657549; doi:10.1371/journal.pone.0241854)
Supplement: S1 Table — Tissue thickness was measured on the day of collection for each core using Vernier calipers following established methods [94]. Mean coral extension for each core was determined as the mean of the top five years of the coral growth analysis (i.e., 2010–2015) to compare the most recent overlapping growth intervals. Core skeletal densities were determined as the mean skeletal density for the entire cored material to account for variations in skeletal densities between skeletal components. Mean calcification rates for each core were determined as the product of the most recent 5-year mean extension rate and mean core skeletal density [9, 11–14]. Mean annual linear extension, skeletal density, and calcification rates were computed for the entire cored skeletal material to provide context for future physiological studies and carbonate production estimates. (DOCX) [file pone.0241854.s003.docx]

**S1 Table.**

| Species | Collection Site | Tissue  Thickness (mm) | Linear  Extension (cm yr^–1^) | Skeletal Density  (g cm^–3^) | Annual  Calcification (g cm^–2^ yr^–1^) | Cores  (n) |
| --- | --- | --- | --- | --- | --- | --- |
| *D. labyrinthiformis* | All Cores | 8.3 ± 1.0 | 0.32 ± 0.06 | 1.37 ± 0.15 | 0.44 ± 0.07 | 15 |
|  | Gurnet Rock | 8.5 ± 0.5 | 0.31 ± 0.07 | 1.43 ± 0.19 | 0.43 ± 0.05 | 3 |
|  | Halfway Flat | 8.3 ± 0.6 | 0.31 ± 0.05 | 1.52 ± 0.06 | 0.47 ± 0.07 | 3 |
|  | Hog Reef | 7.5 ± 1.3 | 0.34 ± 0.05 | 1.23 ± 0.12 | 0.41 ± 0.03 | 3 |
|  | Three Hill Shoals | 8.0 ± 1.7 | 0.30 ± 0.02 | 1.38 ± 0.13 | 0.41 ± 0.06 | 3 |
|  | Whalebone Bay | 9.0 ± 0.0 | 0.37 ± 0.11 | 1.27 ± 0.06 | 0.47 ± 0.14 | 3 |
| *O. franksi* | All Cores | 5.0 ± 0.7 | 0.21 ± 0.05 | 1.85 ± 0.16 | 0.39 ± 0.09 | 15 |
|  | Gurnet Rock | 4.5 ± 0.5 | 0.24 ± 0.05 | 1.75 ± 0.08 | 0.42 ± 0.06 | 3 |
|  | Halfway Flat | 5.5 ± 0.9 | 0.18 ± 0.02 | 2.02 ± 0.21 | 0.37 ± 0.05 | 3 |
|  | Hog Reef | 4.5 ± 0.5 | 0.19 ± 0.07 | 1.90 ± 0.17 | 0.36 ± 0.17 | 3 |
|  | Three Hill Shoals | 5.3 ± 0.6 | 0.23 ± 0.03 | 1.81 ± 0.12 | 0.41 ± 0.06 | 3 |
|  | Whalebone Bay | 5.0 ± 0.9 | 0.22 ± 0.06 | 1.77 ± 0.09 | 0.40 ± 0.13 | 3 |
| *P. strigosa* | All Cores | 8.9 ± 1.0 | 0.38 ± 0.09 | 1.47 ± 0.08 | 0.56 ± 0.11 | 12 |
|  | Gurnet Rock | 9.0 ± 1.0 | 0.39 ± 0.11 | 1.45 ± 0.12 | 0.56 ± 0.12 | 3 |
|  | Hog Reef | 8.0 ± 1.0 | 0.32 ± 0.12 | 1.53 ± 0.05 | 0.49 ± 0.19 | 3 |
|  | Three Hill Shoals | 9.2 ± 0.8 | 0.43 ± 0.05 | 1.40 ± 0.05 | 0.60 ± 0.09 | 3 |
|  | Whalebone Bay | 9.5 ± 0.9 | 0.39 ± 0.04 | 1.48 ± 0.04 | 0.58 ± 0.05 | 3 |
